# Soil-free bioassays for testing novel control agents against *Phytophthora cinnamomi* root rot

**DOI:** 10.3389/fpls.2026.1766319

**Published:** 2026-06-17

**Authors:** Leny Jane Pame, Kenneth G. Pegg, Neena Mitter, Bernard J. Carroll, Louise S. Shuey, Anne Sawyer

**Affiliations:** 1 School of Chemistry and Molecular Biosciences, The University of Queensland, St Lucia, QLD, Australia; 2 Queensland Department of Primary Industries, Ecosciences Precinct, Dutton Park, QLD, Australia; 3 Queensland Alliance for Agriculture and Food Innovation, Centre for Horticultural Science, The University of Queensland, St Lucia, QLD, Australia; 4 Charles Sturt University, Wagga Wagga, NSW, Australia

**Keywords:** exogenous dsRNA, hydroponics, lupin, phosphonate, *Phytophthora cinnamomi*, pineapple, RNA-based biopesticides, SIGS

## Abstract

*Phytophthora cinnamomi* is considered as one of the world’s worst plant pathogens, infecting about 5,000 plant species including those of agricultural and environmental significance. Disease management is largely dependent on chemical control, particularly synthetic fungicides such as phosphonic acid-based fungicides, e.g., phosphite/potassium phosphonate. While phosphonic-acid-based fungicides have been highly effective for more than 40 years, their prolonged use has led to the development of tolerance and decreased sensitivity in *P. cinnamomi*. Novel control agents that are effective but environmentally sustainable are therefore urgently needed. RNA-based biopesticides, which use exogenously applied double-stranded RNA (dsRNA) specific to the target pest or pathogen to avoid off-target effects on other organisms in the environment including beneficials, have emerged as a potential novel disease management strategy against *P. cinnamomi*. Due to the limited availability of bioassays to study the efficacy of this novel control agent against *P. cinnamomi*, we developed water-based lupin and pineapple bioassays using readily available plastic cups and glassware with mycelial plugs as inoculum. Infection rate was assessed 3 to 7 days post-inoculation (dpi) for lupin and 7 to 14 dpi for pineapple by measuring root lesion length and rating root rot. Potassium phosphonate (Agri Fos 600) and dsRNA were tested as example control agents, with dsRNA uptake tested via northern blotting. The bioassays were found suitable for *P. cinnamomi* pathogenicity assays, with one mycelial plug an effective inoculum; fungicide sensitivity testing, with doses as low as 0.45 g L^-1^ Agri Fos® 600 providing protection; and exogenous dsRNA studies targeting root pathogens, with dsRNA able to be taken up by germinating lupin seeds. Overall, the assays are soil-free and thus overcome dsRNA stability issues in the soil and enable the collection of intact clean roots for molecular analyses. Furthermore, the bioassays are non-destructive, allowing root lesion symptoms to be visually monitored and repeatedly measured across different timepoints.

## 1 Introduction

*Phytophthora cinnamomi* Rands is a destructive soilborne oomycete pathogen which causes significant yield losses in horticultural crops and dieback of native plants worldwide. *P. cinnamomi* has been listed as one of the top 100 invasive pathogens worldwide (Burgess et al., 2017) and is prevalent in European nurseries and forests (Jung et al., 2016) and natural ecosystems in South Africa (Von Broembsen, 1985; Nesamari et al., 2017), California (Swiecki et al., 2003), and Australia (DCCEEW Australia, 2025). In Australia, *P. cinnamomi* is a significant threat to 4,000 native species, as well as crops such as avocado, macadamia, and pineapple (Hardham and Blackman, 2018; Queensland Government 2025; DCCEEW Australia, 2025). *P. cinnamomi* infection is driven by motile biflagellate zoospores, which, under favorable conditions (high soil moisture, mild temperature, and neutral to acidic pH), are released from sporangia produced by resting spores and swim to the elongation zone of plant roots, where they encyst (Hardham, 2005; Hardham and Blackman, 2018). The cyst then germinates to produce a germ tube that grows into the roots. Mycelia spread through the root cortex into the central vascular bundle, blocking the transport of water and nutrients in the plant and thus leading to chlorosis and wilting of the leaves and even plant death. Necrosis of fine feeder roots causes dieback and can also quickly lead to death (Hardham and Blackman, 2018).

Current control measures to combat *P. cinnamomi* include the application of fungicides such as phosphite and metalaxyl (Rohrbach and Schenck, 1985; Hardy et al., 2001; Hu et al., 2010) alongside breeding for resistance, e.g. in macadamia (Jeff-Ego et al., 2021), American chestnut (Clark et al., 2023), and avocado (Muthuvel et al., 2025) and other integrated disease management (IDM) strategies (Ramírez-Gil and Morales-Osorio, 2020). However, synthetic control agents can pose harmful effects to the environment due to runoff (Hardy et al., 2001) and toxicity to humans and non-target organisms, including beneficial organisms (Lykogianni et al., 2021), and prolonged use can lead to the development of tolerance or decreased sensitivity of the pathogen (Dobrowolski et al., 2008; Hunter et al., 2023). Breeding for resistance takes considerable time and money and is not feasible in natural environments. Furthermore, the emergence of more virulent races of *P. cinnamomi* is likely—for example, the presence of two mating types (A1 and A2) in South Africa, together with evidence of recombination, may facilitate the emergence of highly virulent and well-adapted lineages of *P. cinnamomi* (Engelbrecht et al., 2022). Other approaches such as the use of biocontrol agents have limitations in terms of limited availability on the market and challenges for registration and commercial development (Palmieri et al., 2022).

Exogenous double-stranded RNA (dsRNA) has emerged as a novel sustainable crop protection platform that is pathogen-specific and does not require any modification to the plant nor leave harmful residues in the environment (Dalakouras et al., 2024; Fletcher et al., 2020). The approach involves the application of pathogen-specific dsRNA to host plants to trigger RNA interference (RNAi) in the invading pathogen (Wang and Jin, 2016; Mosquera et al., 2025). This leads to silencing of essential pathogen genes, inactivating the pathogen and preventing infection. Exogenous dsRNA has great potential for the management of *Phytophthora* diseases and reduction of synthetic fungicide usage (Kalyandurg et al., 2021; Cheng et al., 2022; Sundaresha et al., 2022; Ivanov and Golubeva, 2023; Park et al., 2023) but has yet to be demonstrated against *P. cinnamomi*.

To study the effectiveness of novel control agents such as dsRNA against *P. cinnamomi*, simple, robust, and versatile bioassays are required. A number of bioassays have been developed to screen fungicide efficacy against *P. cinnamomi* and have been carried out on lupin seedlings grown on filter paper/moist paper towel/plastic sheets (Gunning and Cahill, 2009; Stasikowski et al., 2012; Allardyce et al., 2012; Gunning et al., 2013; Eshraghi et al., 2014; Groves et al., 2015), avocado seedlings in a greenhouse (Belisle et al., 2019), and pineapple plants under glasshouse and field conditions (Rohrbach and Schenck, 1985; Anderson et al., 2012). Soil-free bioassays offer greater potential for the study of soilborne pathogens such as *P. cinnamomi* than soil-based assays as they are non-destructive, allowing roots to be easily examined across multiple timepoints without being damaged. This is in contrast to soil systems where it takes time to remove and clean roots without damaging them.

Here we developed soil-free bioassays for *P. cinnamomi* to test the efficacy of synthetic fungicides as well as an exogenous dsRNA targeting *P. cinnamomi translation elongation factor 1-α* (*PcTEF*), which we previously identified as a promising target to control myrtle rust (Degnan et al., 2023a). Lupins were selected as the main host in this study as they are highly susceptible to *P. cinnamomi*, have rapid and uniform growth, are easy to handle, and root lesions are easy to see on the white roots. Indeed lupins are regularly utilized in baiting techniques for *Phytophthora* isolation, identification, and quantification from the soil (Chee and Newhook, 1965; Pratt and Heather, 1972; Eden et al., 2000; Drenth and Sendall, 2001; Burgess et al., 2021).

Our bioassays differ from previously developed *P. cinnamomi* bioassays as they allow the seedlings to be pre-treated with control agents in vermiculite or water, and mycelial plugs are used as the inoculum instead of zoospores, which are difficult to produce for *P. cinnamomi*. Moreover, these bioassays use plastic cups and glass beakers which are cheap and can be re-used. The bioassays are suitable for *P. cinnamomi* pathogenicity assays, fungicide sensitivity testing, and exogenous dsRNA studies targeting root pathogens, circumventing the problem of dsRNA instability in the soil (Dubelman et al., 2014; Bachman et al., 2020; Qiao et al., 2021). Indeed we observed promising dsRNA uptake and persistence in lupin seedlings germinated in vermiculite, warranting further investigation into dsRNA delivery to seedlings via seed treatments. Furthermore, the major advantage of these bioassays is that they are non-destructive, with root lesions able to be visually monitored and measured across different timepoints using a simple ImageJ workflow. Soil-free and clean roots can also be easily harvested for further molecular analyses.

## 2 Materials and methods

### 2.1 Plant materials and pathogen isolates

Open-pollinated seeds of New Zealand blue lupins (*Lupinus angustifolius*) were purchased from The Lost Seed Company (Tasmania, Australia). Crowns of pineapple (*Ananas comusus*) hybrids 73–50 and MD-2 were provided by Valley Syndicate (Bungundarra, Australia) and Fullerton Farms (Elimbah, Australia).

Two *Phytophthora cinnamomi* isolates, BRIP-70497 (pineapple) and an avocado isolate, were provided by Dr. Ken Pegg (Queensland Department of Primary Industries, QDPI) and maintained as water cultures. BRIP-70497 was isolated from the soil from a pineapple farm in Beerwah, Queensland by Mr. Neil Parami and Dr. Ken Pegg and is conserved in the Queensland Plant Pathology Herbarium. The *P. cinnamomi* avocado isolate was isolated by Dr. Ken Pegg. *P. cinnamomi* cultures were sub-cultured and grown on either 15% or 20% V8 (clarified Campbell’s V8 vegetable juice, 1% CaCO_3_) agar and maintained at room temperature (RT) in the dark.

### 2.2 Soil-free water-based lupin bioassays in clear plastic cups

**Figure 1:**
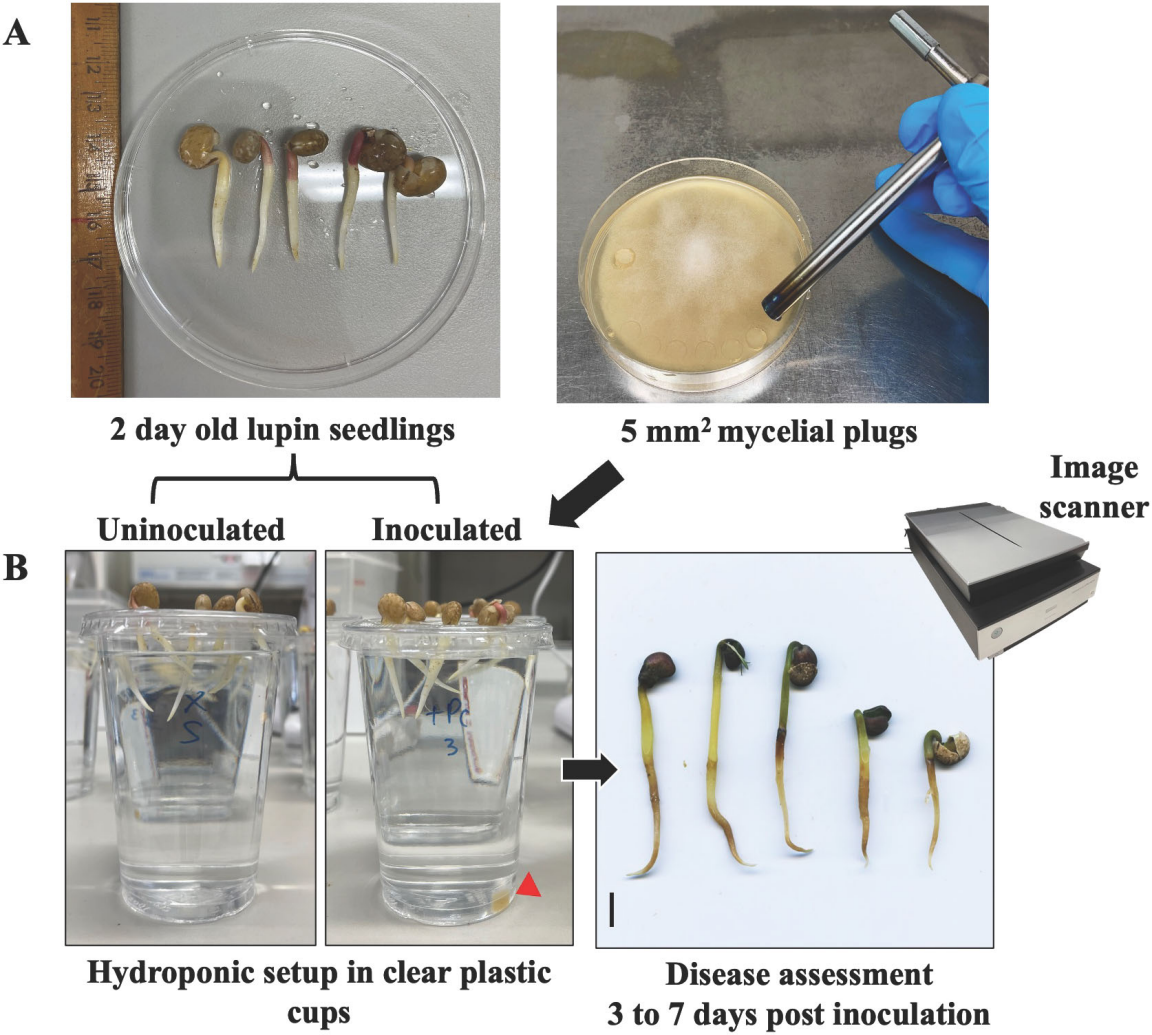
*Phytophthora cinnamomi* soil-free bioassay set up for lupins. **(A)** Two-day-old lupin seedlings are germinated in vermiculite before being placed into plastic cups containing ~275 mL of water; these are then inoculated with one 5-mm^2^ mycelial plug from a 4–7-day-old *P. cinnamomi* culture grown on 15% V8 agar. Mycelial discs are created using a metal cork borer. **(B)** Plastic cups with five seedlings placed in each plastic lid hole showing the uninoculated and inoculated seedlings (one 5-mm^2^ mycelial plug, red arrowhead). Root lesions are assessed 3 to 7 days post-inoculation using a scanner. Scale bar = 1 cm.

Lupin seeds were surface-sterilized in 1% bleach for 5 min, rinsed with reverse osmosis (RO) water at least four times to remove excess bleach, and then soaked in MilliQ^®^ water for 10 min to facilitate water imbibition into the seeds. Surface-sterilized lupin seeds were sown in wet vermiculite in medium (22 cm × 25 cm) to large (27 cm × 35 cm) transparent resealable plastic bags with a 2:1 ratio of vermiculite to water and grown on a lab bench at RT under overhead lights set on a 12/12-h day photoperiod for 2 days. At 2 days post-sowing, the lupin seedlings were rinsed in RO to remove the debris, and seedlings with 2–4-cm-long roots were selected (**Figure 1A**).

For the water-based bioassay setup, 295-mL (10 oz) clear plastic cups (STATPAK Stationery and Packaging) with clear lids containing five holes were filled with approximately 275 mL of MilliQ^®^ water, and five germinated lupin seedlings were transplanted to each lid hole. For inoculation, one 5-mm^2^ *P. cinnamomi* avocado isolate mycelial plug from actively growing 4- to 7-day-old cultures was directly added into the water (**Figure 1B**). The seedlings were incubated at RT (12/12-h day photoperiod) until disease assessment. Infection rate (% root lesion length) was assessed 3 to 7 days post-inoculation by measuring the length of roots with and without lesions with ImageJ (**Supplementary Figure 1**). The lupin seedlings were imaged using an Epson Perfection V700 flatbed scanner.

### 2.3 Phosphonate treatment of lupins *via* drench application

Agri Fos® 600 systemic fungicide (600 g L^-1^ phosphorous acid present as mono- and di-potassium phosphonate salts, Agrichem®), which is also known as potassium phosphonate (KP), was added into the vermiculite with surface-sterilized lupin seeds according to the following dilution rates: 0.1×, 0.01×, 0.005×, 0.0025×, 0.001× (1× = 4.5 g L^-1^ Agri Fos 600 or 8.25 mL of Agri Fos 600 per liter of water, which is the standard rate for foliar sprays of avocado in the field) in a 2:1 ratio of vermiculite to water.

### 2.4 Double-stranded RNA treatment of lupins *via* drench application

A total of 1 to 2 mg of *P. cinnamomi translation elongation factor 1-α* (*PcTEF*) dsRNA or *YBR219C* dsRNA (non-specific control targeting yeast) (Genolution, South Korea; see **Supplementary Table 1** for sequences) was mixed into 125 mL of water containing 0.1 mM EDTA and 0.01% Pulse penetrant (1,000 g L^-1^ polyether-modified polysiloxane; NuFarm, Australia) and directly applied into the vermiculite (2:1 ratio of vermiculite and dsRNA solution) with 50 surface-sterilized lupin seeds.

### 2.5 Root lesion assessment

For both lupin bioassays, infection rate (% root lesion length) was measured using ImageJ version 1.54g. This was calculated by measuring the total length of the lupin root from the root node to the root tip and the lesion length and dividing the lesion length by the total root length. A step-by-step procedure for root length measurement in ImageJ is shown in **Supplementary Figure 1**. Plant fresh weight was an added parameter in the plastic cup bioassay. For this, five pooled lupin seedlings from each biological replicate were pat-dried on a paper towel and then weighed using a digital benchtop weighing scale.

### 2.6 Soil-free pineapple bioassays in glass containers

**Figure 2:**
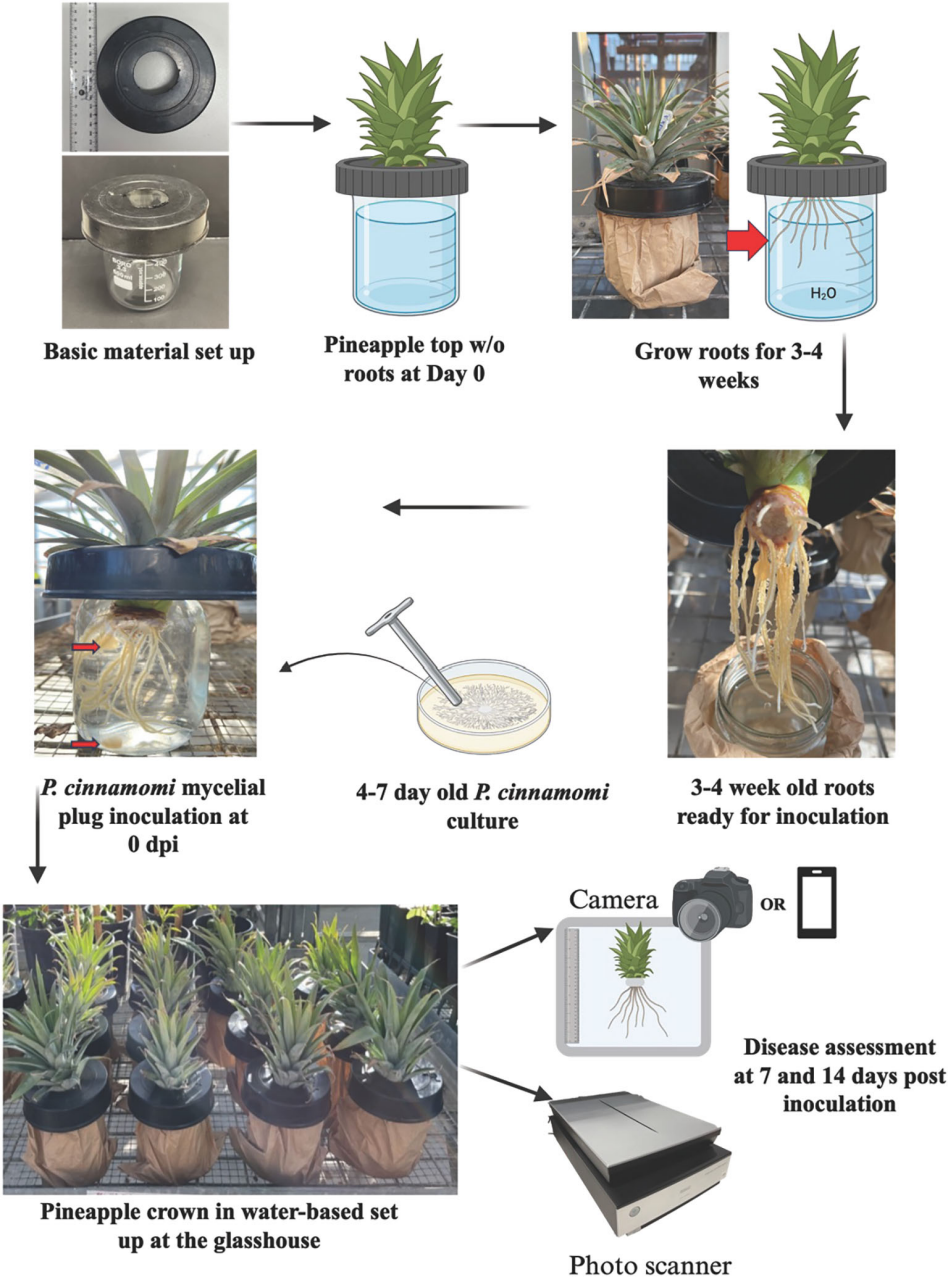
*Phytophthora cinnamomi* soil-free bioassay setup for pineapple tops with emerging roots. Schematic diagram of the basic experimental workflow of the *P. cinnamomi* pathogenicity assays on pineapple roots in water-based bioassay using reverse osmosis (RO) water in 500-mL glass beakers covered in brown paper (top panel). The roots are usually ready for inoculation 3 to 4 weeks after transplanting. For inoculation, one 10-mm^2^ *P. cinnamomi* mycelial plug is added to the water; the other is inserted in between the roots (middle panel). The pineapple roots are then grown in hydroponics under controlled conditions, and disease symptoms were observed 7 and 14 days post-inoculation (dpi). Photographs can be taken using either a mobile phone, digital camera, or image scanner (bottom panel). Some of the images in the diagram were created using BioRender.com.

**Figure 3:**
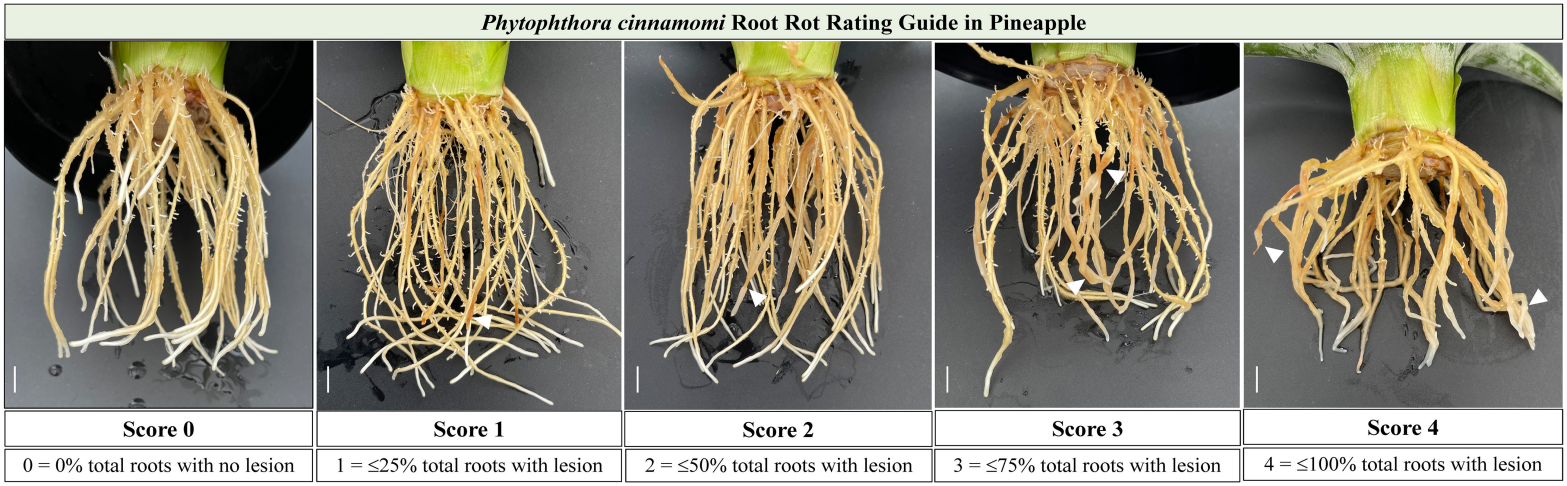
*Phytophthora cinnamomi* root rot rating guide for pineapple pathogenicity assays. This root rot rating guide of *Phytophthora cinnamomi*-infected pineapple roots provides a scoring system for phenotypic symptoms ranging from 0 to 4 with a corresponding percentage of the total number of roots with lesions. This guide can be used for pineapple roots grown in hydroponics or water-grown pineapple during disease assessment timepoints between 7 and 21 days post-inoculation. White arrowheads indicate pineapple root lesions. Scale bar = 1 cm.

The pathogenicity assay on pineapple was carried out in 500-mL glass beakers or jars covered with brown paper bags and fitted with 13-cm-diameter lids with holes in the middle (**Figure 2**, top panel). The pineapple crowns were thinly cut to expose the fresh butt and then sprayed with 70% ethanol to prevent mold infection prior to transplanting. The crown butts were cured in a butt-up position for at least 2 to 3 days prior to transplanting. Upon transplanting, the glass containers were filled with 500 mL of RO water, and one crown was transplanted per beaker. The pineapple roots were grown in RO water for about 3 to 4 weeks in the glasshouse under natural light, with an average temperature of 24°C–27°C and relative humidity of 60%–70% (**Figure 2**, top panel). Water was replenished weekly due to the absence of an aeration system. At 3 to 4 weeks post-transplanting, the pineapple roots were inoculated with 10-mm^2^ mycelial plugs, with at least one plug inserted in between the roots and the rest directly added to the water (**Figure 2**, middle panel). The crowns continued to grow in the glasshouse, and disease symptoms were scored at 7 and 14 days post-inoculation (**Figure 2**, bottom panel) following our *P. cinnamomi* root rot rating guide for pineapple (**Figure 3**). Images were captured and documented with a mobile camera and Epson Perfection V700 flatbed scanner.

### 2.7 Phosphonate treatment of pineapple roots in water

KP was added to the water according to the desired dilution rates (0.001×, 0.01×, 0.05×, and 0.1×) based on the standard rate for foliar sprays of avocado in field application (1× = 4.5 g L^-1^ Agri Fos 600 or 8.25 mL per liter of water) 24 h prior to inoculation. At 24 h after KP application, the water was replaced with 500 mL of fresh RO water.

### 2.8 Double-stranded RNA uptake by lupin seeds in vermiculite

750 μg of *green fluorescent protein* (*GFP*) dsRNA (Genolution, South Korea) was added to surface-sterilized lupin seeds (three seeds per 50 mL Falcon tube) in vermiculite ± 0.01% Pulse and 0.1 mM EDTA. The ratio was 2:1 vermiculite (10 mL volume) to dsRNA/water (5 mL). The seeds were germinated for 48 h in dsRNA. Seedlings were collected and washed with MilliQ^®^ water to reduce the likelihood of surface residues (i.e., dsRNA or vermiculite), and the seed coat was removed from the cotyledon. The samples were snap-frozen in liquid nitrogen prior to RNA extraction and northern blot analysis.

### 2.9 RNA extraction and northern blot analysis

Total RNA extraction from lupin tissue was carried out using TRIzol^TM^ reagent following the manufacturer’s instructions (ThermoFisher Scientific) or a Monarch Total RNA miniprep kit (New England Biolabs) following the tough-to-lyse sample protocol.

Extracted total RNA (1 to 5 μg) was separated using formaldehyde denaturing gel electrophoresis and then transferred onto a positively charged Hybond-N+ nylon membrane (GE Healthcare) via capillary transfer overnight. After transfer, the membrane was probed with a digoxigenin (DIG)-labeled (Roche) *GFP* DNA probe in ULTRAHyb™ Ultrasensitive Hybridization Buffer (ThermoFisher Scientific) at 42°C for 24 h, and the DIG-labeled RNA was detected using anti-digoxigenin-AB Fab fragments (Roche) and CDP-star chemiluminescent substrate (Applied Biosystems). An Amersham Ai800 imaging system (Cytiva) was used for visualization.

### 2.10 Statistical analysis

Data analysis was performed using GraphPad Prism version 10 for MacOs using ordinary one-way ANOVA with Tukey’s multiple comparison test (*P* < 0.05) and unpaired *t*-test (two-tailed, *P* < 0.05).

## 3 Results and discussion

### 3.1 Establishment of a soil-free lupin bioassay

To study the pathogenicity of *Phytophthora cinnamomi* and its response to novel control agents, an improved soil-free lupin bioassay was developed using readily available plasticware (**Figure 1**). The bioassay was based on the protocol described by Drenth and Sendall (2001), which was a modification of one originally developed by Chee and Newhook (1965). The main difference here, however, is that our bioassay is soil-free and mycelial plugs were used as inoculum rather than *Phytophthora-*infected soil or zoospores. This was due to difficulties in being able to routinely produce sterile sporangia and zoospores using published approaches such as the mineral salt solution method described by Chen and Zentmyer (1970). Sporangia could be easily produced by incubating mycelial plugs in pond water; however, this led to contamination with other microbes and was thus not suitable for these assays. Mycelial plugs were therefore used as inoculum here as these readily produced sporangia and zoospores when placed into the plastic cups with the lupin seedlings.

One mycelial plug was found to be a consistently effective inoculum for the successful infection of 2-day-old lupin seedlings, with primary disease symptoms, i.e., lesions on roots visible as early as 3 days post-inoculation (dpi) and peaking at 7 dpi (**Figure 1**, **Supplementary Figure 2**). Secondary symptoms were visible at 7 dpi and included wilted leaves/cotyledons and the absence of leaf emergence (**Figure 1**, **Supplementary Figure 2**). Successful infection was evident by the presence of hyphae on the root tip (zone of elongation) and intercellular and intracellular hyphal penetration (**Supplementary Figure 3**). Moreover, sporangia were observed on the mycelial plugs 7 dpi. To confirm *P. cinnamomi* pathogenicity and fulfill Koch’s postulates, infected roots were plated on *Phytophthora* selective medium (CMA P5ARP).

### 3.2 Potassium phosphonate is an effective control against *Phytophthora cinnamomi* on lupins grown in water

To further examine the versatility of the soil-free lupin bioassay, different concentrations of potassium phosphonate (Agri Fos® 600) were tested to determine its efficacy in protecting lupin roots from *P. cinnamomi* infection in water. Potassium phosphonate (KP) has been widely used for the management of *Phytophthora* diseases on various horticultural crops (Pegg et al., 1985; Rohrbach and Schenck, 1985; Pegg et al., 1987, 1990; Guest et al., 1994, 1995; Vawdrey et al., 2004; Browne and Viveros, 2005; Anderson et al., 2012; Nyoni et al., 2021) and native plants (Aberton et al., 1999; Jackson et al., 2000). It has a suppressive mode of action based on the stimulation of host defense responses and a direct fungistatic effect on oomycete pathogens (Guest and Grant, 1991). The KP used in this study is a salt of phosphonic acid that has been registered as a fungicide in the agriculture sector (Dann and McLeod, 2021).

**Figure 4:**
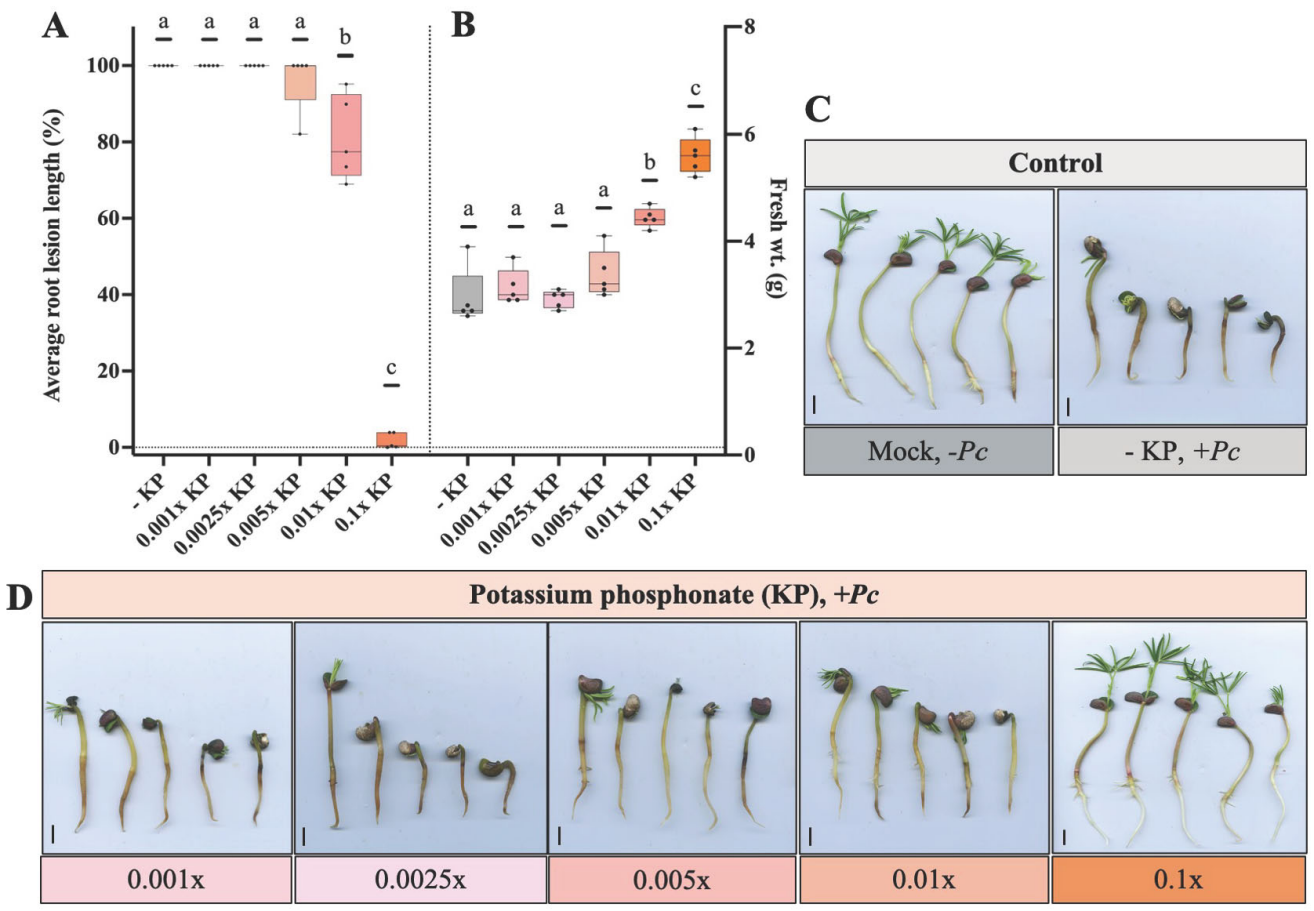
Sensitivity of *Phytophthora cinnamomi* to different levels of potassium phosphonate (KP). Seeds were sown in vermiculite drenched with KP. Two days later, the seedlings were transferred to clear plastic cups containing 275 mL of MilliQ® water and inoculated with one *P. cinnamomi* (avocado) mycelial plug. **(A)** Infection rate (average root lesion lengths in %) and **(B)** fresh weights of inoculated lupin seedlings (+*Pc*) with different concentrations of KP 7 days post-inoculation (dpi). The treatments are as follows: uninoculated as negative control; no potassium phosphonate (-KP) as positive control; 0.1× KP, 0.01× KP, 0.005× KP, 0.0025× KP, and 0.001× KP dilution rates as fungicide-treated treatment groups. The root lesion lengths were measured using ImageJ version 1.53q, whereas the seedlings were weighed on a digital bench weighing scale. **(C, D)** Scanned images of the treatments applied with and without KP 7 days post-inoculation using an Epson Perfection V700 flatbed scanner. Treatment groups 0.1× and 0.01× KP were repeated in more than three independent experiments, while 0.001× was repeated twice and the rest was only tested once. Graphs were plotted using GraphPad Prism 10. Whiskers represent minimum to maximum values of the biological replicates (comprising a group of five seedlings/subsamples per biological replicate). Treatments with the same letter above denote no statistical difference according to an ordinary one-way ANOVA (Tukey’s multiple comparison test), alpha = 0.05. Scale bar = 1 cm.

To determine the effect of different concentrations of KP on lupin root infection, seeds were germinated in vermiculite soaked in 0–0.1× KP (1× being the standard application rate of 8.25 mL per liter) prior to inoculation. We explored seed treatment as it is often utilized as a delivery mechanism for pesticides and to prevent seedborne diseases. 0.1× KP was used as the highest concentration (**Figure 4**), as preliminary tests showed that 1× KP was phytotoxic to lupins in a water-based system, causing soft white root rot and root thinning (**Supplementary Figure 4**, upper panel). Further evidence of KP phytotoxicity at high concentrations is provided in the study of Jee et al. (2002) on the effect of KP on *Phytophthora* root rot (PRR) in hydroponic lettuce. The authors found that the addition of 500 ppm of KP in a hydroponic system slightly restricted the growth of lettuce but not when less than or equal to 200 ppm KP was applied, indicating that lower amounts of KP are required for water-based or hydroponic settings. To date, there is no available information on the concentration effect of KP in water-based versus soil-based bioassays. However, under field conditions, there is evidence that drenching of pineapple hearts with 0.5% w/v of Agri Fos® 600 as a post-plant treatment can retard growth compared to when the crowns were pre-plant dipped (Anderson et al., 2012), suggesting that high concentrations of KP cause phytotoxicity when applied directly to the roots and other soft or vulnerable plant tissues regardless of the growing conditions.

The results clearly showed that 0.1× KP mediated a statistically significant protective effect compared to lower concentrations (**Figures 4A, D**) and the -KP inoculated control (**Figures 4A, C**). This outcome was also confirmed by root fresh weights, with roots of 0.1× KP-treated seedlings being significantly heavier than the roots of seedlings treated with the other concentrations and comparable with the uninoculated control (**Figure 4B**). Moreover, no phytotoxicity symptoms were observed at 0.1× KP. Taken together, this result shows that the plastic cup bioassay can be used for fungicide sensitivity screening against *P. cinnamomi*.

One issue that was noted with the plastic cup setup was that uninoculated lupin roots developed some browning on roots with purpling hypocotyl tissue (**Figure 4C**, **Supplementary Figure 5**). In the pilot setup, seedlings were grown under constant light to induce sporangia production as light exposure has been found to be crucial during the sporangia induction stage (Pegg, pers communication; Del Castillo-González et al., 2024). However, it was suspected that constant light exposure may have caused these symptoms as a similar phenotype was previously observed by Yokawa et al. (2014). To test this hypothesis, one set of seedlings was grown under a lamp that was continuously on and another under ceiling lights that were switched off at night. While the seedlings grown under the lamp displayed considerable root browning and purpling on the hypocotyl and upper part of the roots, the seedlings grown without the lamp showed little to no root browning (**Supplementary Figure 5**). These results show that it is important to optimize light conditions in lupin bioassays to avoid non-pathogen-induced root browning. In the successive bioassays, a lamp was therefore set up approximately 60–80 cm above the plants, with a light timer set to a 12-h day/12-h night cycle to minimize non-pathogen root browning.

**Figure 5:**
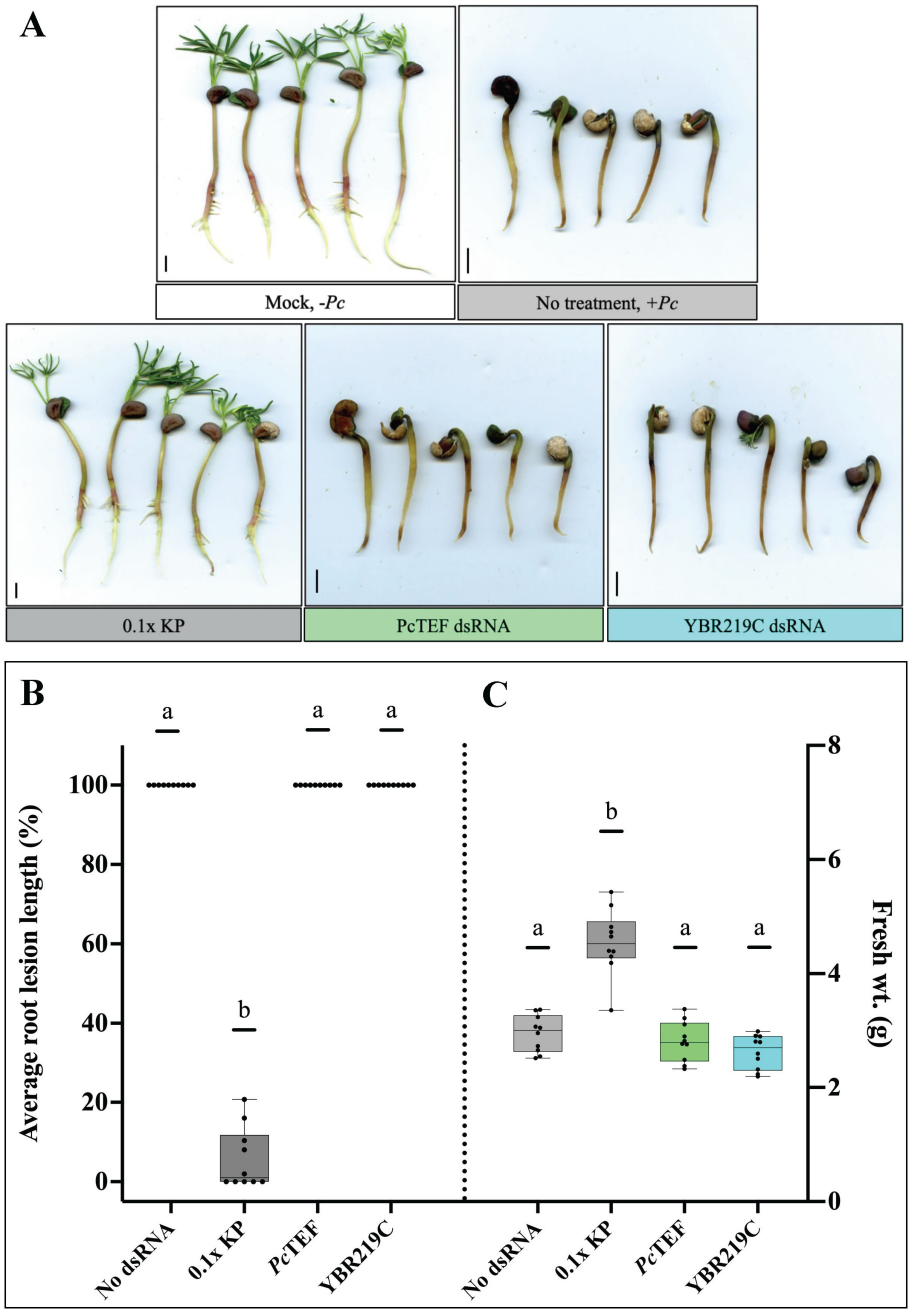
Exogenous dsRNA treatment of lupin seeds *via* drench application in vermiculite. Two treatment groups were evaluated for dsRNA-mediated protection of lupins against *P. cinnamomi* following a drench application of dsRNA for 48 h in vermiculite. The treatments included 1 mg of *PcTEF* dsRNA (targeting *P. cinnamomi translation elongation factor* 1-α) and 1 mg of *YBR219C* (non-specific dsRNA targeting yeast). The controls included mock (uninoculated), inoculated (no treatment with dsRNA or potassium phosphonate (KP)), and 0.1× KP treatment. Moreover, 0.1 mM EDTA and 0.01% Pulse penetrant were added to each treatment group. Bioassays were carried out in clear plastic cups containing 275 mL of MilliQ® water, with 10 cups containing five seedlings per biological replicate. Each cup was inoculated with one *P. cinnamomi* (avocado) mycelial plug except for the mock cup which was inoculated with one sterile V8 (-Pc) plug. **(A)** Scanned images of the treatments applied with and without dsRNA and KP 7 days post-inoculation using an Epson Perfection V700 flatbed scanner. **(B)** Root lesion lengths were measured using ImageJ version 1.53q. **(C)** The seedlings were weighed on a digital bench weighing scale. This result was confirmed in a repeat experiment. Graphs were plotted in GraphPad Prism 10. Whiskers represent minimum to maximum values of the biological replicates. Treatments with the same letter above denote no statistical difference according to an ordinary one-way ANOVA (Tukey’s multiple comparison test), alpha = 0.05. Scale bar = 1 cm.

Interestingly, we found that addition of one sterile V8 plug without *P. cinnamomi* in the mock/uninoculated control prevented browning (**Supplementary Figure 6**); therefore, V8 plugs were included in the uninoculated (mock) control in the dsRNA lupin bioassay experiment (**Figure 5A**). It is also worth noting that lupin roots of uninoculated (mock) controls grown in a pipette box set up with a 12-h day/12-h night light cycle did not show any browning (**Supplementary Figure 4**, upper left panel). This may imply that in addition to optimal light conditions, provision of nutrients to the roots (e.g., from V8) and different types of containers (i.e., recycled orange pipette tip box) may also solve the issue of browning of lupin roots in water-based bioassays.

### 3.3 Soil-free pineapple bioassay for *P. cinnamomi*

A second soil-free bioassay was explored for the *P. cinnamomi*–pineapple pathosystem. It was first investigated whether pineapple and avocado *P. cinnamomi* isolates could infect the roots of two pineapple hybrids, MD2 and 73-50. A *P. cinnamomi* root rot rating guide for pineapple was developed to assess root health. The scores ranged from 0 to 4, with 0 for when all roots were healthy, 1 when ≤25% of roots had lesions, 2 when ≤50% of roots had lesions, 3 when ≤75% of roots had lesions, and 4 when all of the roots had lesions (**Figure 3**). The pineapple root rot rating has an interval of 25% in each score based on lesion symptoms in roots where the % root lesions were qualitatively estimated.

**Figure 6:**
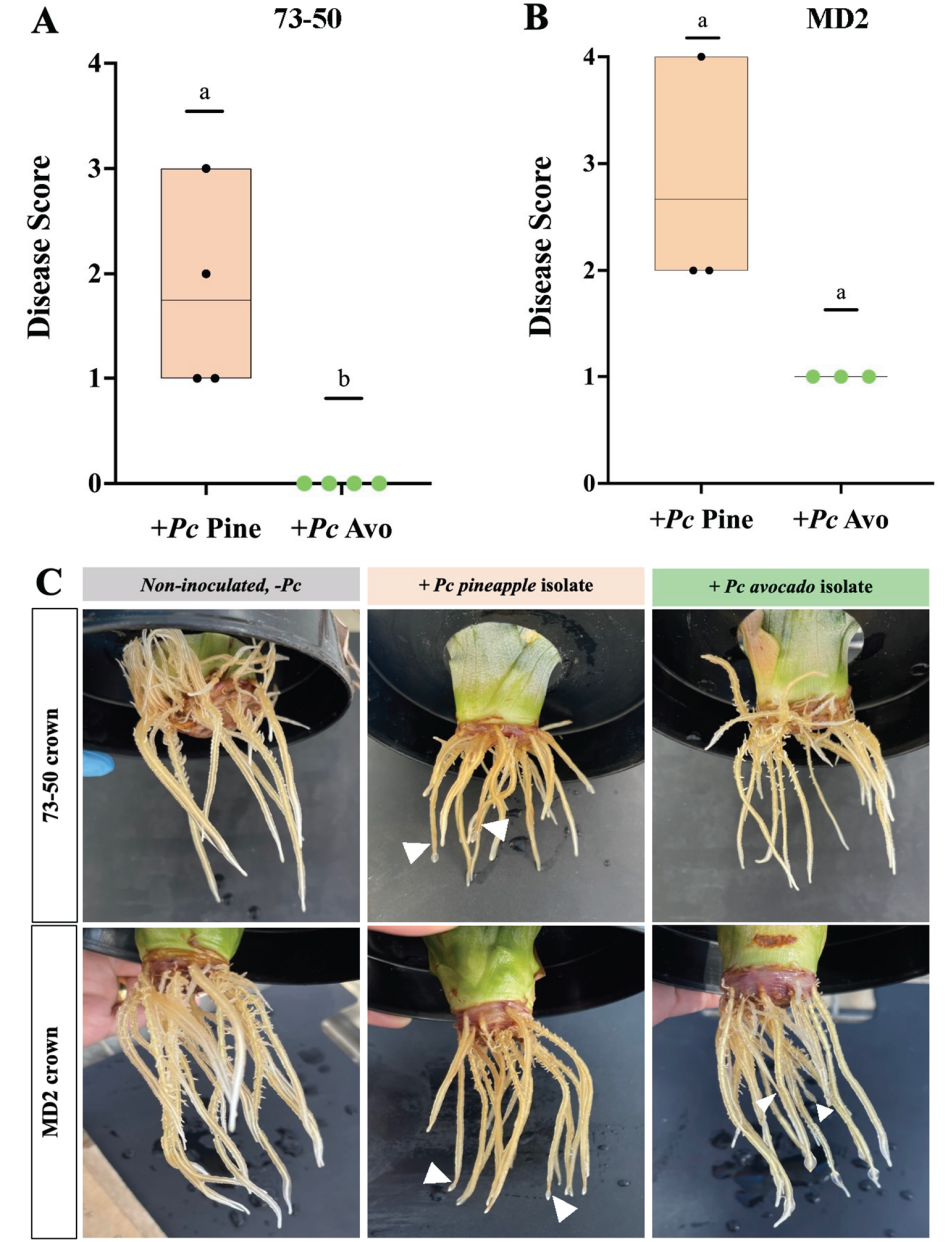
*Phytophthora cinnamomi* pathogenicity assay on 4-week-old pineapple roots. Extent of infection of **(A)** 73–50 and **(B)** MD2 roots by two *P. cinnamomi* isolates (pineapple and avocado) inoculated with four (MD2 roots) or five (73–50 roots) 10-mm^2^ mycelial plugs. The following disease scoring system was used: 0 = 0% total roots with no lesion or healthy, 1 = ≤25% total roots with lesion, 2 = ≤50% total roots with lesion, 3 = ≤75% total roots with lesion, and 4 = ≤100% total roots with lesion. The graphs were plotted in GraphPad Prism 10. Floating bars represent minimum and maximum values of the replicates (dots), and the mean value is indicated by a line within the bar. Treatments with the same letter above denote no statistical difference according to an unpaired *t*-test (two-tailed, *P* < *0.05*). **(C)** Photographs were taken 7 days post-inoculation using a mobile phone camera. White arrowheads indicate pineapple root lesions.

The pineapple isolate caused considerable root rot on both pineapple hybrids, while the avocado isolate only caused symptoms on MD2 roots (**Figure 6**). It is worth noting that the 73–50 pineapple hybrid was more tolerant to root rot (**Figures 6A, C**—top panel) compared to MD2, even when the inoculum level was increased from four to five mycelial plugs (**Figures 6B, C**—lower panel). This supports the results of Anderson et al. (2012) who reported that MD2 is more susceptible to *P. cinnamomi* heart rot than 73-50. Therefore, the failure of the avocado isolate to infect pineapple hybrid 73–50 is not particularly surprising. Inoculating the pineapple roots with two pineapple isolate mycelial plugs instead of five was also explored. The results revealed that two mycelial plugs were sufficient inoculum source for pineapple root infection, with symptoms observed as early as 7 days post-inoculation (**Supplementary Figure 7**). In summary, a water-based pathogenicity assay using mycelial plugs as inoculum is feasible for the *P. cinnamomi*–pineapple pathosystem.

**Figure 7:**
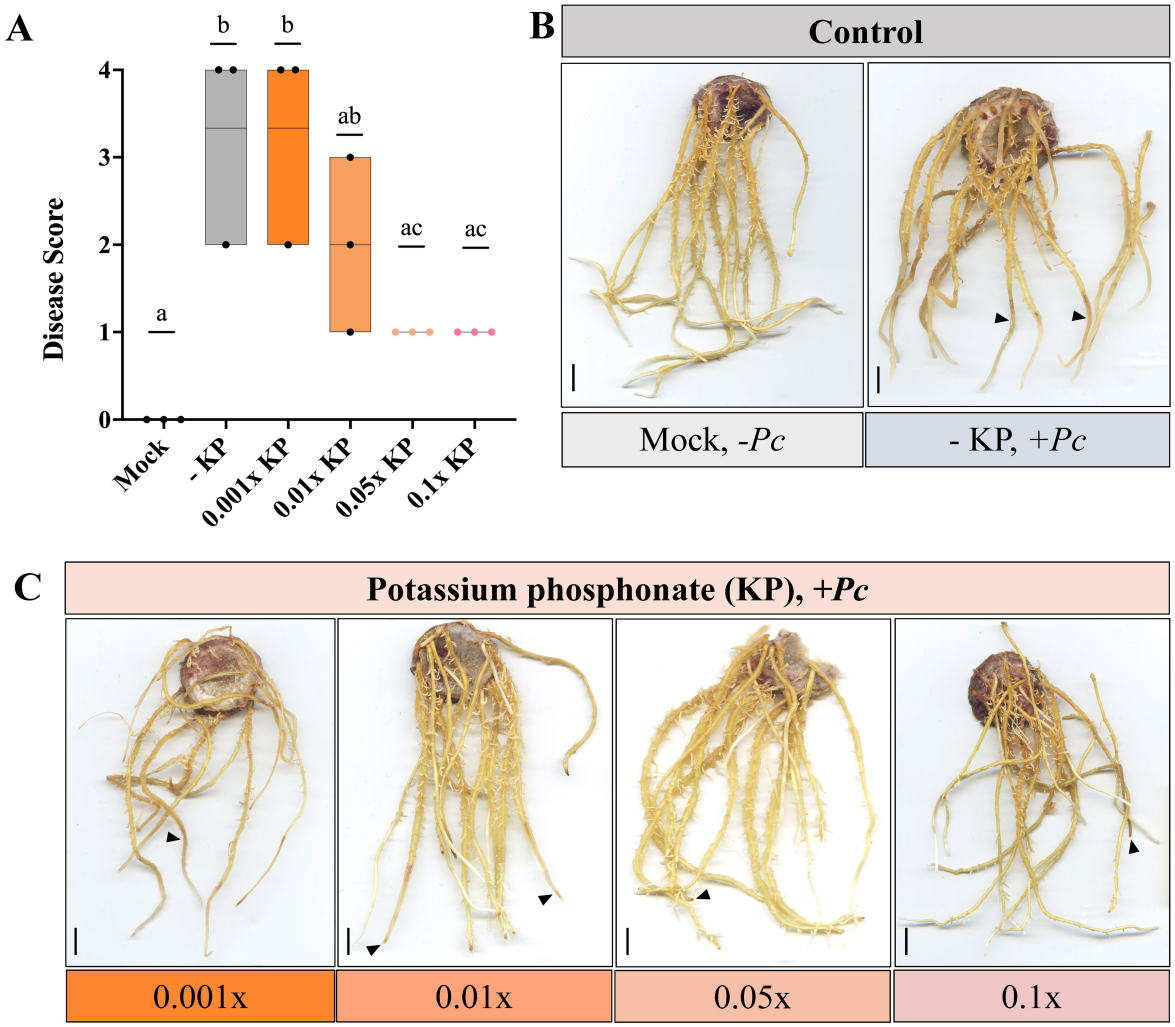
*Phytophthora cinnamomi-*fungicide sensitivity assay on 4-week-old pineapple roots. Pineapple hybrid 73–50 was grown in 500 mL of reverse osmosis (RO) water in a glass beaker/jar and inoculated with two mycelial plugs of *P. cinnamomi pineapple* isolate at 4 weeks post-transplanting into hydroponics. Different concentrations of potassium phosphonate (KP) were added to the water 24 h before inoculation. The treatment groups (three biological replicates per treatment group) were as follows: 0.1× KP, 0.5x KP, 0.01× KP, and 0.001× KP dilution rates as fungicide-treated treatment groups, uninoculated as a negative control, and no KP as a positive control. **(A)** Infection rates of pineapple roots following the disease scoring system: 0 = 0% total roots with no lesion or healthy, 1 = ≤25% total roots with lesion, 2 = ≤50% total roots with lesion, 3 = ≤75% total roots with root lesion, and 4 = ≤100% total roots with lesion. Scanned images of the **(B)** treatment groups without KP and **(C)** with KP 14 days post-inoculation were taken using Epson Perfection V700 flatbed scanner. Black arrowheads indicate pineapple root lesions. Graphs were plotted in GraphPad Prism 10. Floating bars represent minimum and maximum values of the replicates (dots), and the mean value is indicated by a line within the bar. Treatments with the same letter above denote no statistical difference according to an ordinary one-way ANOVA (Tukey’s multiple comparison test), alpha = 0.05. Scale bar = 1 cm.

The pineapple bioassay was next utilized to test the efficacy of different concentrations of KP. In pineapple, KP has been shown to reduce heart and root rot caused by *P. cinnamomi* as well as base rot caused by the ascomycete fungus *Ceratocystis paradoxa* (Anderson et al., 2012, 2022). In this study, different concentrations of KP were directly applied to the water where the pineapple roots were growing 24 h prior to inoculation. The results indicated that 0.1× and 0.05× KP significantly mediated partial protection with an average disease score of 1 (≤25% roots with lesions, **Figure 3**) compared to 0.001× KP and the -KP control (**Figure 7**). Note that the high variability in disease scores between biological replicates in treatment groups (-KP, 0.001× KP, and 0.01× KP; **Figure 7A**) could be due to the low number of biological replicates (three replicates per treatment group). This was due to the limited availability of pineapple crowns. Moreover, the variability of pineapple roots could also be a factor given that the disease score is an estimate of the root lesions (%). Nevertheless, this demonstrates that a water-based pineapple bioassay can also be utilized as a good system to study soilborne pathogens such as *P. cinnamomi* and to test new control agents, but an increased number of biological replicates is needed.

### 3.4 Uptake and persistence of dsRNA in germinating lupins

**Figure 8:**
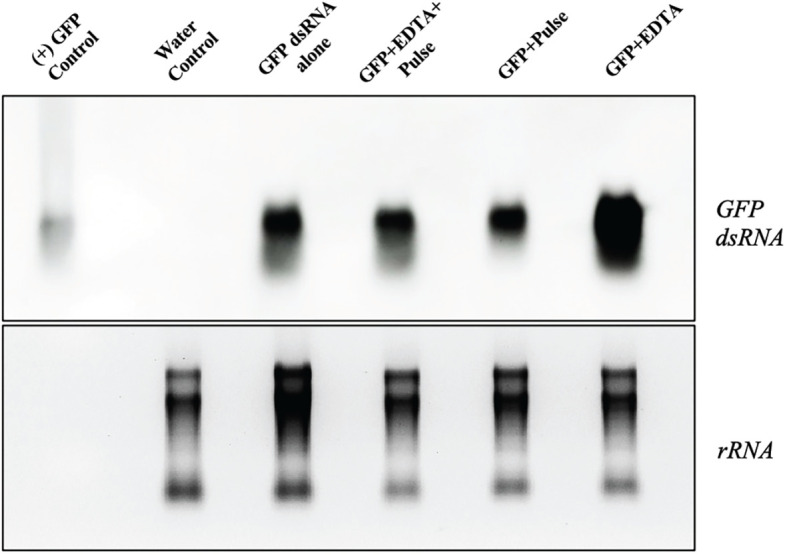
Exogenously applied dsRNA is efficiently taken up by germinating lupin seedlings in vermiculite. In the upper panel is the RNA gel blot probing *GFP* dsRNA uptake in lupin seedlings treated with water as a control, *GFP* dsRNA alone (150 ng/μL), *GFP* dsRNA + 0.01% Pulse + 0.1 mM EDTA, *GFP* dsRNA + 0.01% Pulse, and *GFP* dsRNA + 0.1 mM EDTA 48 h post-application. Ribosomal RNA (rRNA) is shown as a loading control in the lower panel. *GFP* control (+) was 5 ng *GFP* dsRNA. The total amount of RNA loaded per lane was approximately 3 to 5 μg.

To determine whether dsRNA was stable in vermiculite upon seed treatment and able to be taken up by the germinating lupin seeds, lupin seeds were germinated in vermiculite drenched in dsRNA solution, and northern blotting was conducted to detect whether the applied dsRNA was present in the seedlings at 48 h post-application. Pulse penetrant and EDTA were also tested to see whether they improved uptake and prevented dsRNA degradation, respectively. It is also worth noting that the seed coat was removed prior to the collection of seedlings for RNA extraction to ensure that any dsRNA detected came from the internal tissues. Intact dsRNA was present in lupins in all treatment groups at 48 h post-application (**Figure 8**, upper panel), confirming that dsRNA can be absorbed by lupin seeds/seedlings during germination in vermiculite. Although dsRNA could be taken up by the seeds/seedlings without the addition of penetrants or EDTA, based on the brightness of the bands in **Figure 8**, EDTA may still enhance dsRNA uptake and stability, something which will need to be explored further in the future with a more quantitative approach such as qPCR. Nevertheless, these results suggest that exogenous application of dsRNA to vermiculite is an effective delivery method for lupin.

### 3.5 Suitability of soil-free bioassays for assessing dsRNA as a control agent for *Phytophthora cinnamomi*

The lupin bioassay was utilized to test if a dsRNA construct targeting the *P. cinnamomi translation elongation factor* 1-α (*PcTEF*) gene could be used for dsRNA-mediated protection of lupin roots against *P. cinnamomi*. This target previously proved effective in preventing and curing rose apple from myrtle rust disease caused by *Austropuccinia psidii* (Degnan et al., 2023a, 2023b). The dsRNA solution (20 μg per seed) was added directly into the vermiculite and mixed with surface-sterilized seeds. Neither *PcTEF* dsRNA nor a non-specific dsRNA (*YBR219C*) conferred protection to lupin seedlings; however, 0.1× KP treatment resulted in a significant protective effect (**Figure 5A**), consistent with previous results (**Figure 4**). A similar trend was also seen with the fresh weights, where higher fresh weights correlated with a lower infection rate for the KP treatment and lower fresh weights with higher infection rates in the dsRNA-treated and inoculated control (**Figure 5C**). In case the result was dose dependent, a second lupin bioassay was set up with double the dsRNA dose (40 μg per seed). However, *PcTEF* dsRNA still failed to protect the lupin seedlings from *P. cinnamomi* infection (**Supplementary Figure 8**).

The explanation for the failure of *PcTEF* dsRNA to confer protection to lupin seedlings (**Figure 5**; **Supplementary Figure 8**) is unclear, given that uptake and persistence of dsRNA were confirmed in the lupin seedlings (**Figure 8**). Higher concentrations of dsRNA may be required to protect lupins against *P. cinnamomi*. Alternative *P. cinnamomi* target genes will also need to be explored, and dsRNA uptake efficiency by the pathogen will need to be investigated.

## 4 Conclusion

Our soil-free bioassays demonstrate that water-based systems are practical, easy to set up, and suitable for studying soilborne pathogens such as *P. cinnamomi.* These assays are particularly useful for pathogenicity and fungicide sensitivity experiments when the setup requires direct application to the roots and dose optimization of chemical compounds such as new synthetic fungicides, which would be difficult and tedious in a soil-based experiment in the first stage of fungicide screening trials. With the promising dsRNA uptake and persistence seen in lupins germinated in vermiculite, this soil-free bioassay could also be used in exogenous dsRNA studies where the dsRNA solution is applied to vermiculite or water rather than soil substrates in which dsRNA is unstable, and clean roots can be collected without being damaged for molecular analysis. Another practical advantage is that this is a non-destructive bioassay, and root lesion symptoms can be visually monitored and repeatedly measured across different timepoints of interest using a simple ImageJ workflow. Although the bioassay was developed for *P. cinnamomi*, it could be modified for other pathosystems.

## Data Availability

The original contributions presented in the study are included in the article/**Supplementary Material**. Further inquiries can be directed to the corresponding author.
