# Supplementary material for: Soil-free bioassays for testing novel control agents against Phytophthora cinnamomi root rot
Source: Front Plant Sci. 2026 Jun 17;17:1766319. doi: 10.3389/fpls.2026.1766319 (PMC13319037; doi:10.3389/fpls.2026.1766319)
Supplement: Supplementary file 1 [file DataSheet1.pdf]

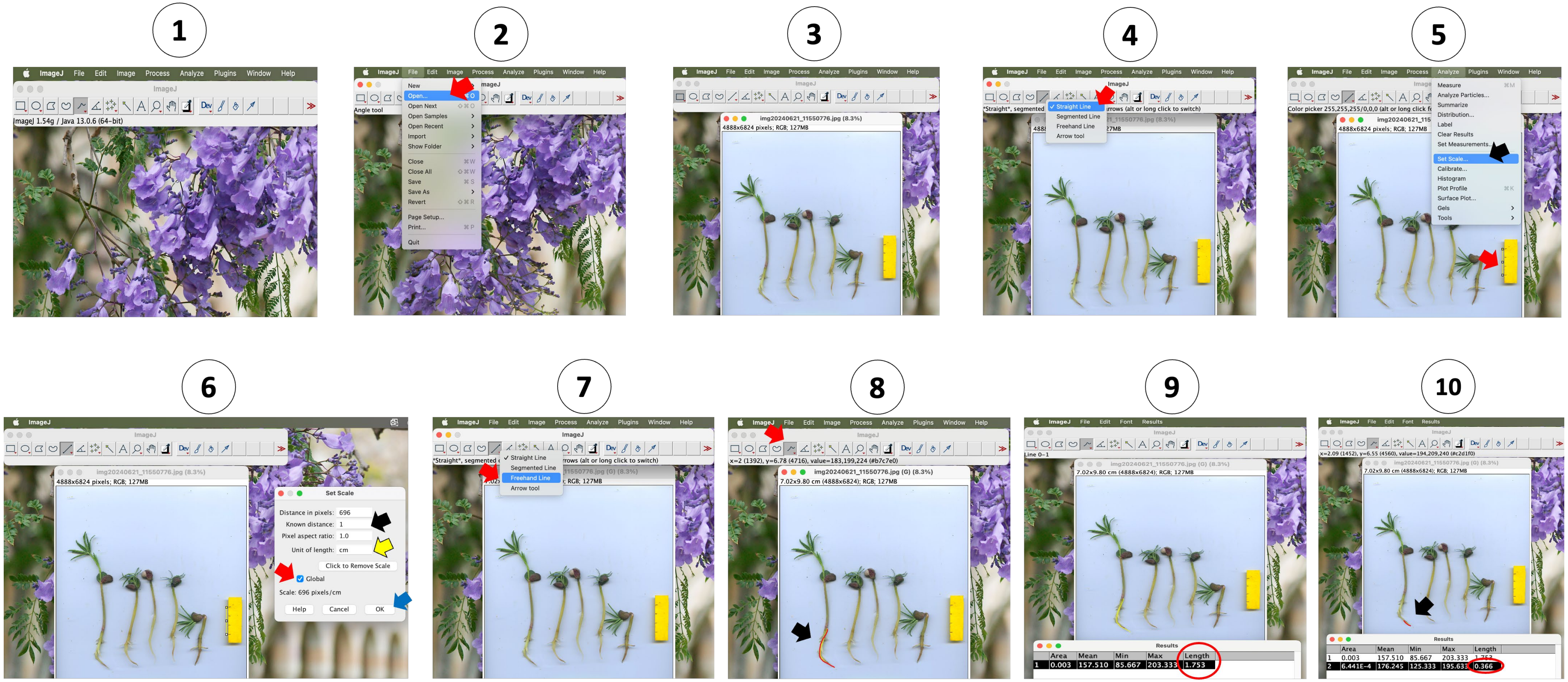

Supplementary Figure 1. Disease assessment of lupins using ImageJ for MacOS. (1) Open ImageJ and the menu bar and tool selections will appear; **(2)** Click 'File' in the menu bar to open an image file; **(3)** The image pops up in the ImageJ window/screen. **(4)** To set a scale for root length measurement, select the 'straight line selection' tool (red arrow). **(5)** First draw a straight line with the ruler/pre-measured scale (red arrow), then click 'Analyze' in the menu bar and select the 'Set scale' (black arrow) to open a new window. **(6)** A new dialog box appears on the left side of the screen to set the scale. Change the settings according to your known distance (black arrow) and unit of length (yellow arrow). When the 'Global'

option is checked, the defined scale is used for all images measured. **(7)** To begin measuring the root, select the 'Freehand line selection' tool first (red arrow), then **(8)** start dragging the black cursor from top to bottom of the roots as indicated by a red line (black arrow) to get the total root length. **(9)** Press shortcut key 'Command+M' or select 'Analyse' in the menu bar and select 'Measure'. A new dialog box appears showing the measurement results that also show the total root length being measured (circled in red). **(10)** Repeat step 9 except only measure the lesion (red line indicated by black arrow), and the dialog box continues to show more results where the lesion length is provided (circled in red).

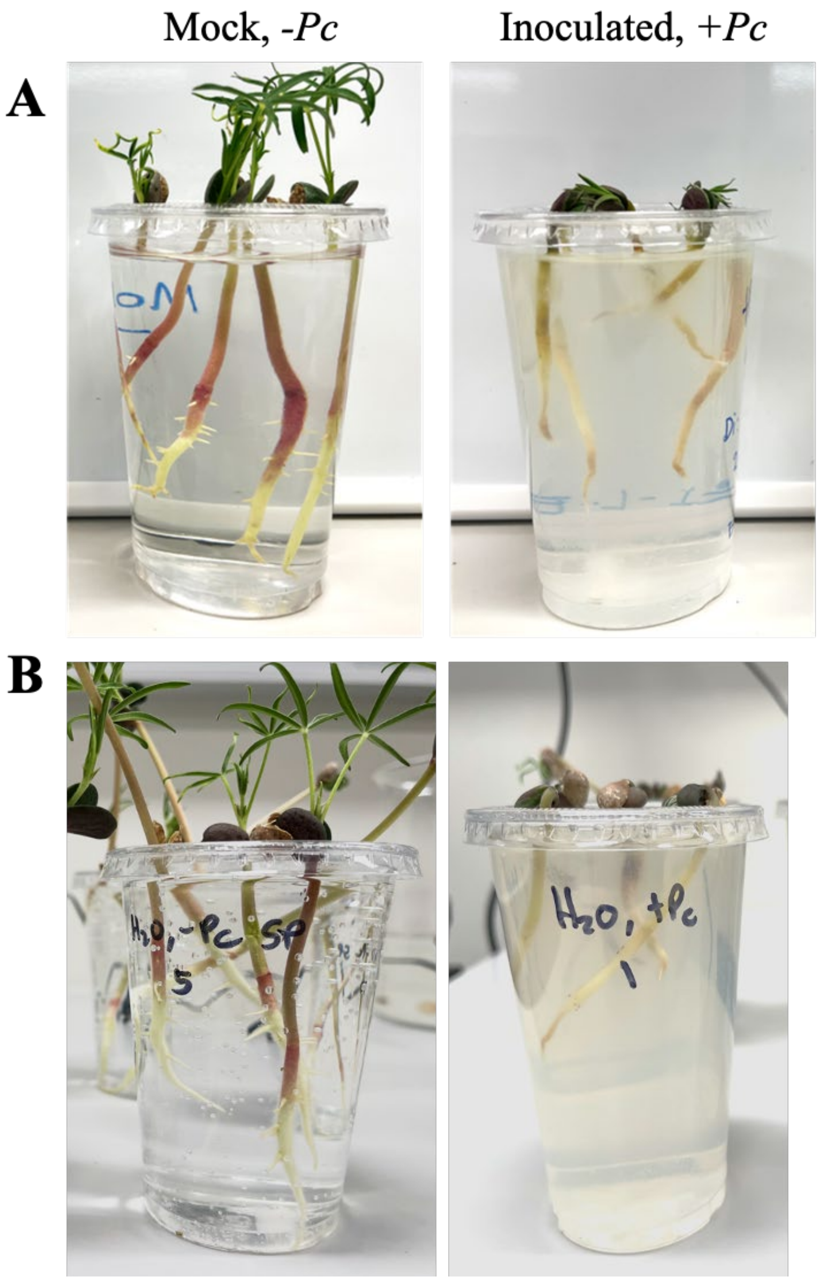

Supplementary Figure 2. *Phytophthora cinnamomi* pathogenicity assays on 2-day old lupin seedlings in a soil-free system. Seedlings were inoculated with one 5 mm^2 mycelial plug of *Phytophthora cinnamomi avocado* isolate (right panel). Uninoculated (mock) controls contained either **(A)** no plug (left panel) or **(B)** a -*Pc* V8 agar plug . Photos were taken 7 days post inoculation.

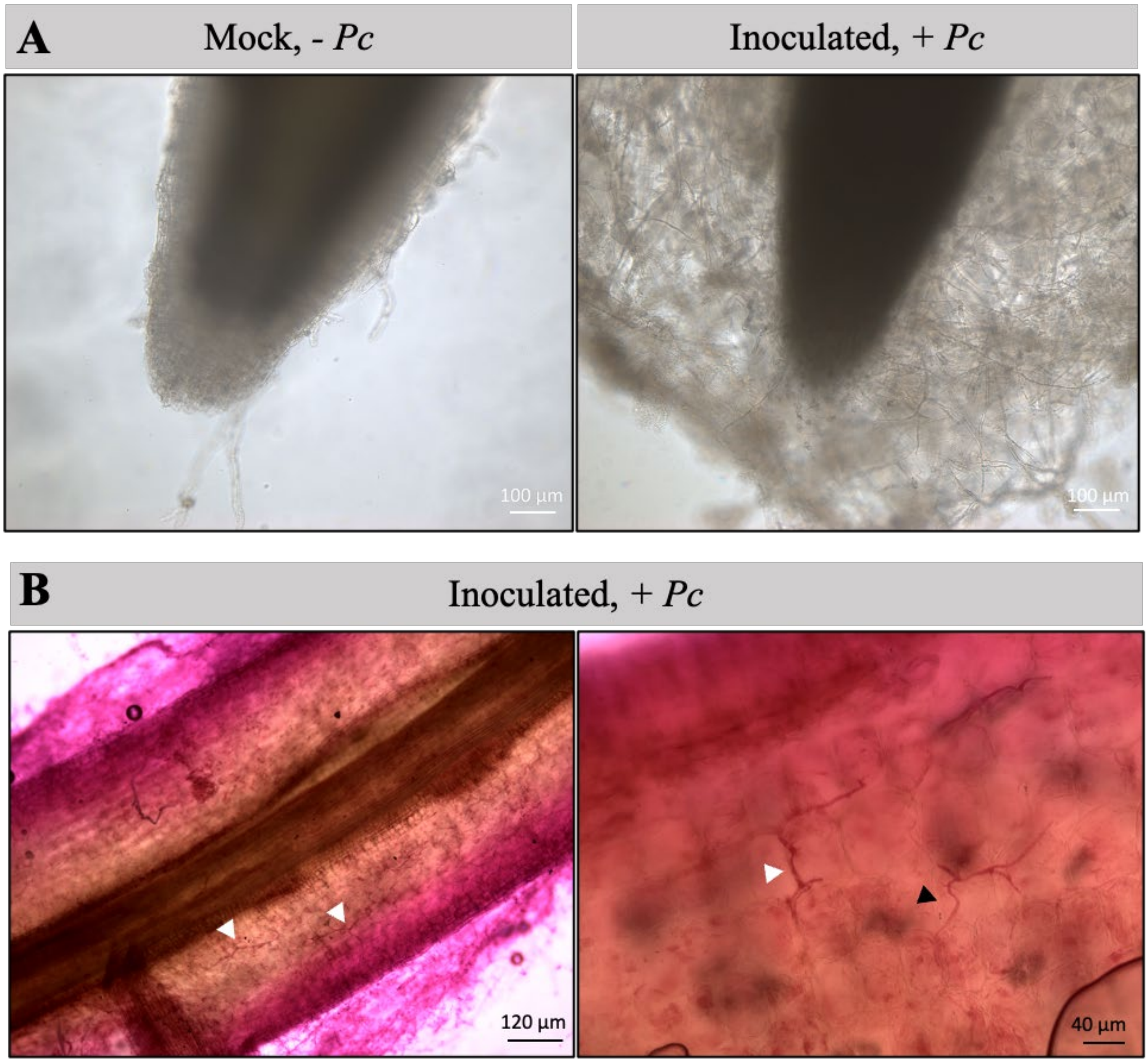

Supplementary Figure 3. *Phytophthora cinnamomi* avocado isolate successfully infects lupin roots. **(A)** Microscopic images of an uninfected root (left panel) and infected root (right panel) with hyphae visible around the root tip. Microscopy images were taken using a ZEISS AxioImager M2 fluorescence microscope. **(B)** Microscopic images of a longitudinal section of lupin root showing successful intercellular (white arrowhead) and intracellular (black arrowhead) hyphal penetration into the root tissue. Roots were stained with lacto-fuchsin staining solution. Microscopy images were taken using an Olympus EP50 digital microscope camera installed on an Olympus CX43 compound light microscope.

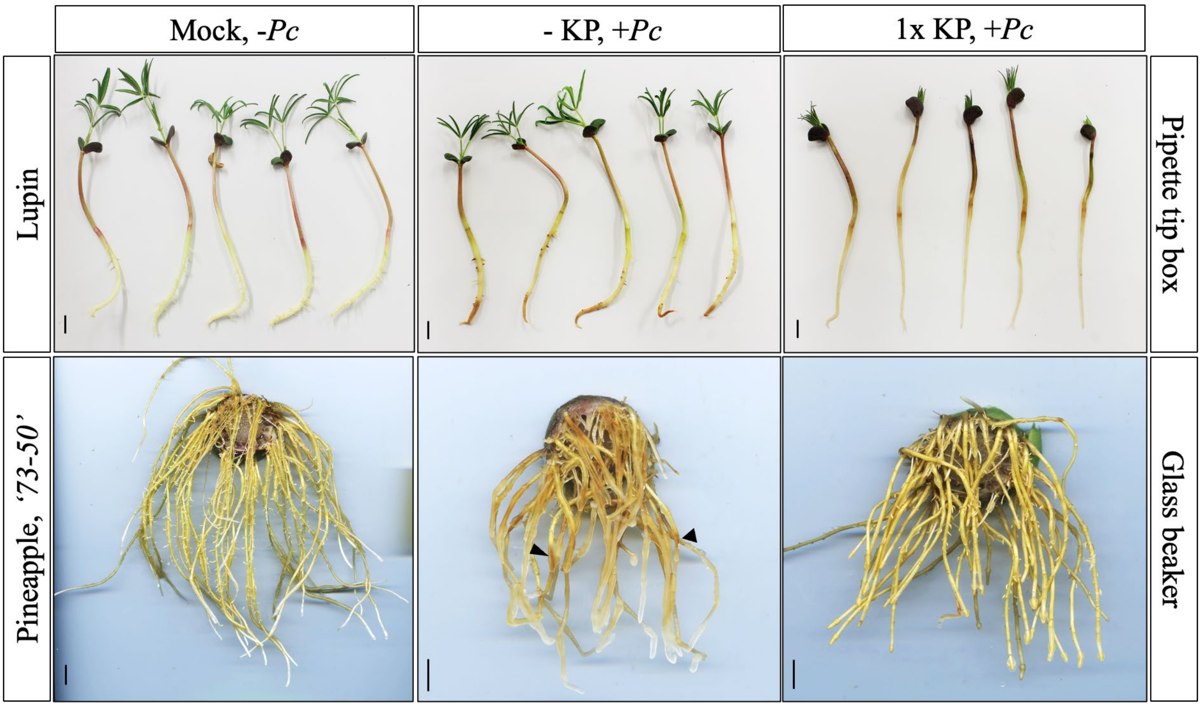

Supplementary Figure 4. Potassium phosphonate application to roots in water. The standard foliar spray rate of potassium phosphonate (KP) for avocado (8.25 ml per litre) was applied directly into ~500 mL of water to lupin roots in a 1 mL pipette tip box 24 hours pre-inoculation and throughout infection **(upper panel)** and The KP was not washed off the entire 7 day inoculation period, whereas in pineapple hybrid *73-50*, KP was only applied 24 h before infection, with the water replaced with fresh water lacking KP immediately before infection **(lower panel)**. Lupins were grown in pipette tip boxes instead of plastic cups while pineapples were grown in glass beakers. **Upper panel** images were taken using a mounted Canon EOS 600D digital camera 7 days post inoculation, whereas **lower panel** images were captured by an Epson Perfection V700 flatbed scanner 14 days post inoculation. Black arrowheads indicate pineapple roots with lesions. Scale bar = 1 cm.

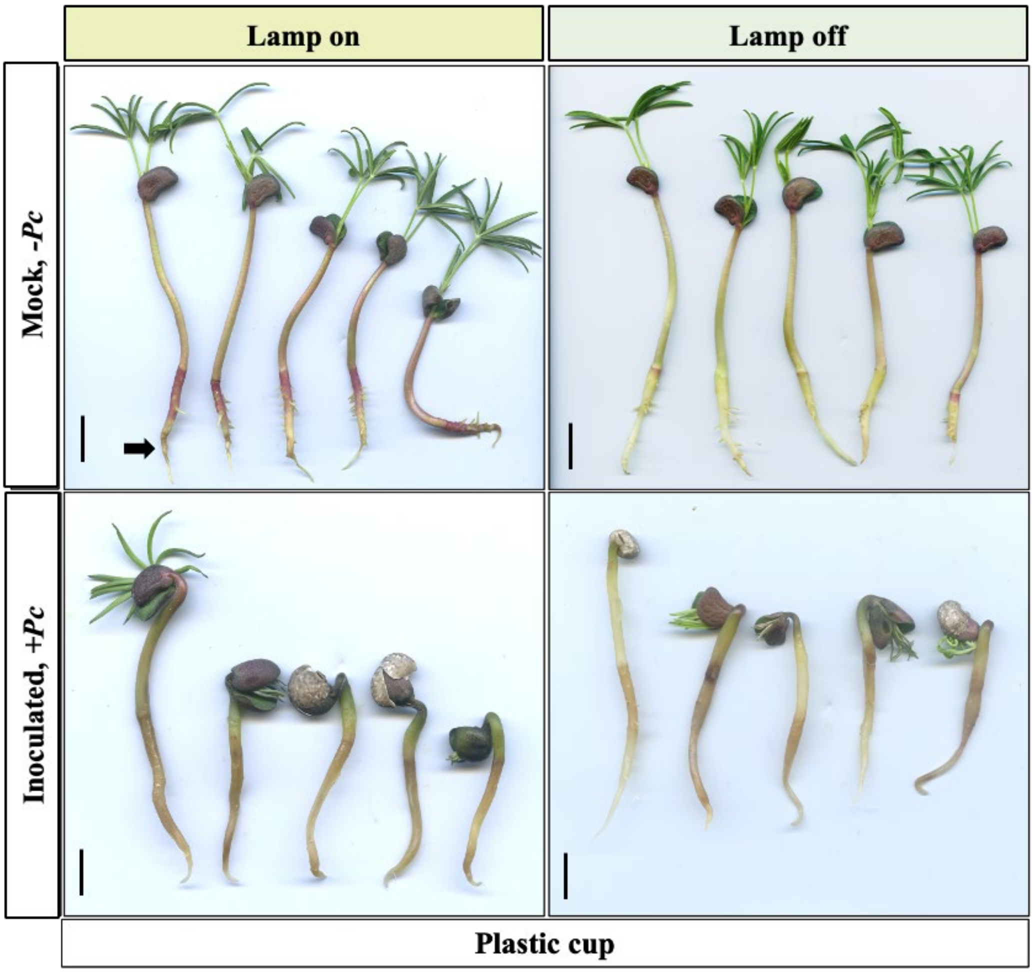

Supplementary Figure 5. Constant exposure of lupin roots to light causes browning. Uninoculated lupin roots ***(Mock, black arrow)*** of two-day old lupin seedlings grown in clear plastic cups with MilliQ® water under constant light exposure developed brown roots **(lamp on, upper left panel)** making them difficult to differentiate from infected seedlings with root lesions **(lower panel)**. All five roots were infected by adding one 5 mm^2 mycelial plug of 7-day old *P. cinnamomi avocado* isolate culture directly into the water in the cup. All treatments were incubated at room temperature. Images were taken 7 days post inoculation using an Epson Perfection V700 flatbed scanner. Scale bar = 1 cm.

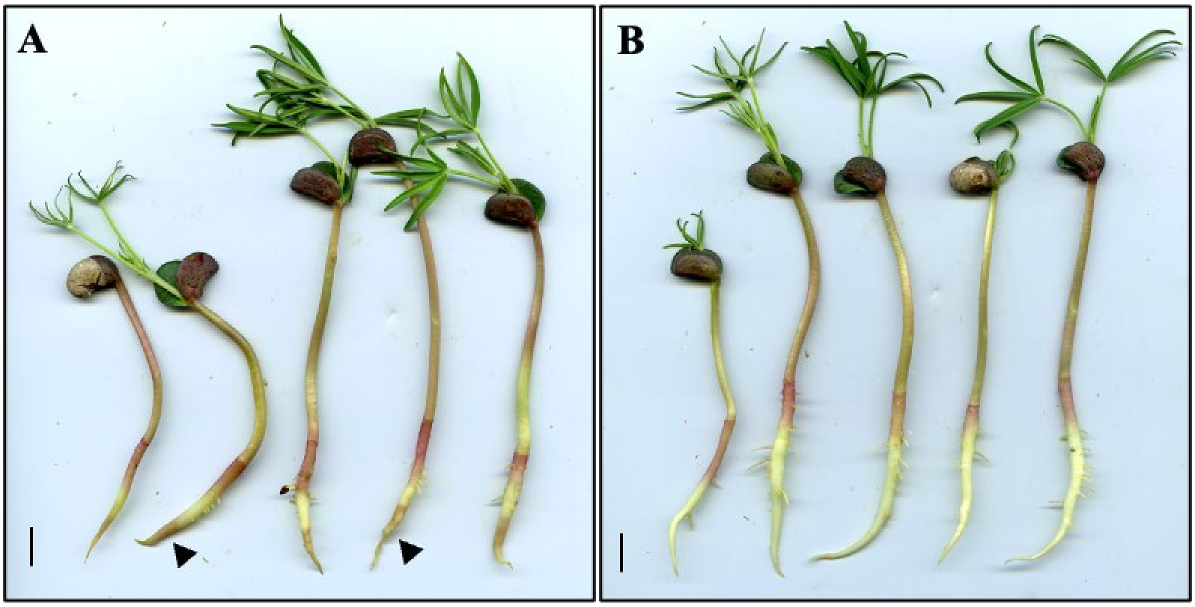

Supplementary Figure 6. Addition of a V8 plug in the uninoculated/mock control prevents browning of lupin roots. (A) Uninoculated control lupin roots without a V8 plug. Black arrows indicate root browning. **(B)** Uninoculated control with one V8 plug. Images were taken 7 days post inoculation using an Epson Perfection V700 flatbed scanner. Scale bar = 1 cm

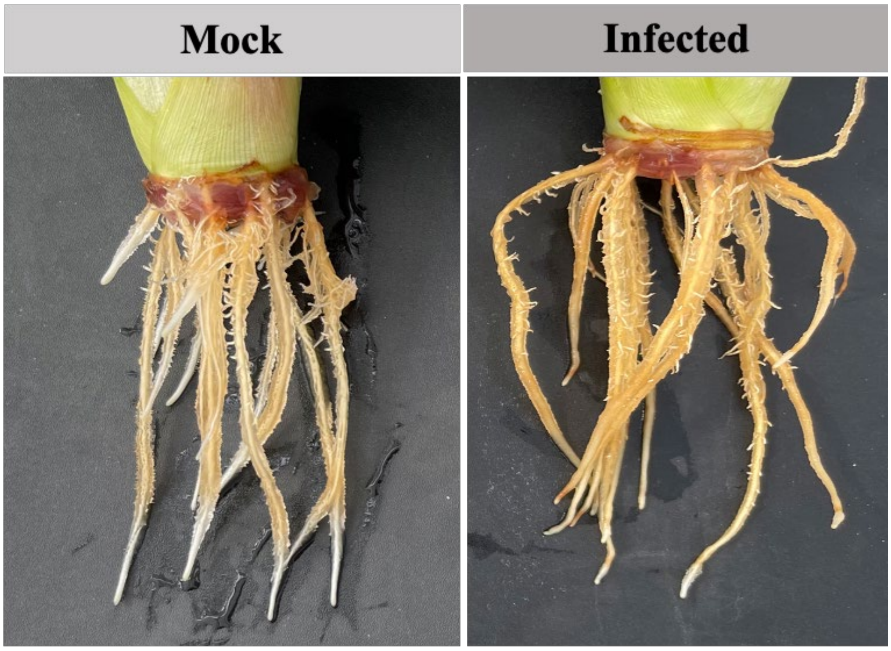

Supplementary Figure 7. Two mycelial plugs of *Phytophthora cinnamomi* pineapple isolate are sufficient for infection of 4-week-old *73-50* pineapple roots. The infected pineapple roots **(right panel)** show the extent of infection 7 days post inoculation compared to a mock, uninfected pineapple **(left panel)**. Photographs were taken 7 days post inoculation using an Iphone 12 Pro Max camera.

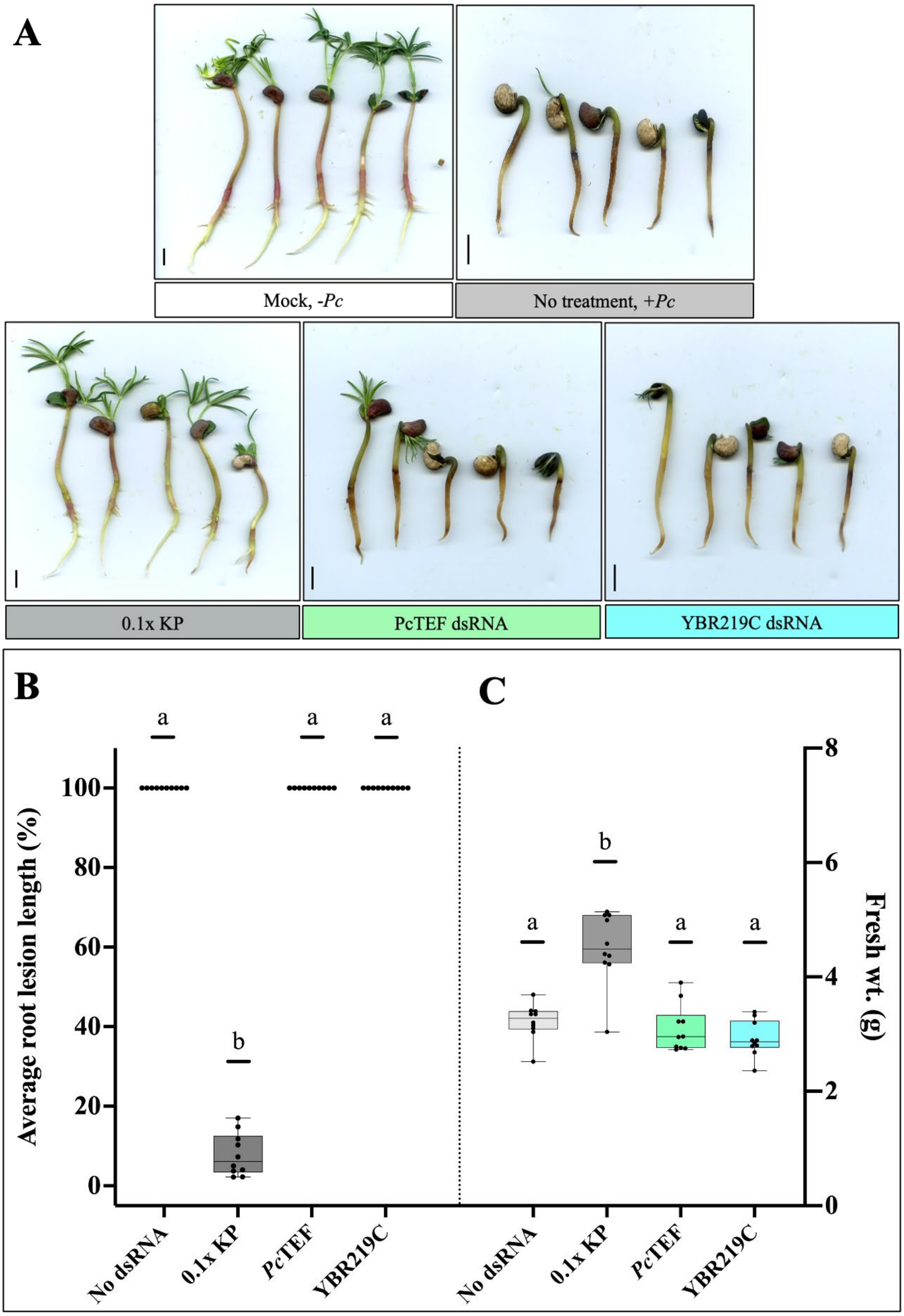

Supplementary Figure 8. Exogenous dsRNA treatment of lupin seeds using double the dsRNA dose (40 µg per seed). Two treatment groups were evaluated for dsRNA-mediated

protection of lupins against *Phytophthora cinnamomi* following drench application of dsRNA for 48 hours in vermiculite. Treatments included 2 mg of *PcTEF* dsRNA (targeting *P. cinnamomi translation elongation factor* 1-α) and *YBR219C* (non-specific dsRNA targeting yeast) in 125 mL of water. Controls included a mock (uninoculated), inoculated (no dsRNA or KP) and 0.1x KP treatment. 0.1 mM EDTA and 0.01% Pulse penetrant were added to each treatment group. Bioassays were carried out in clear plastic cups containing 275 mL of MilliQ® water with 10 cups containing 5 seedlings per biological replicate. Each cup was inoculated with one *P. cinnamomi* (avocado) mycelial plug or for the mock one -Pc plug. **(A)** Scanned images of the treatments applied w/ and w/o dsRNA and KP 7 days post inoculation using an Epson Perfection V700 flatbed scanner. **(B)** Root lesion lengths were measured by ImageJ version 1.53q.**(C)** Seedlings were weighed on a digital bench weighing scale. This result was confirmed in a repeat experiment. Graphs were plotted in GraphPad prism 10. Whiskers represent the min to max values of the biological replicates. Treatments with the same letter above denote no statistical difference according to Ordinary one-way ANOVA (Tukey's multiple comparison test), *alpha = 0.05*. Scale bar = 1 cm.

Supplementary Table 1. dsRNA sequences used in this study.

Description	Length (bp)	Sequence
PcTEF	303	GAAGAAGGUGGGCUACAAGCCGGCCAAGAUCCCGUUCGUGCCCAUCUCCGGCUGGGAGGGCGACAACAUGAUCGAGAAGUCGGGCAACAUG<br>CCGUGGUACAAGGGCCCGUACCUCCUUGAGGCUCUCGACAACCUGAACCCCCCCAAGCGCCCGGUUGACAAGCCGCUGCGUCUGCCCCUCCA<br>GGACGUGUACAAGAUCGGCGGUAUCGGCACGGUACCGGUCGGCCGUGUGGAGACCGGUGUCAUCAAGCCUGGCAUGGUCGCCACGUUCGGC<br>CCCGUGGGUCUGUCGACGGAAGUCAAGUC
YBR219C (Yeast)	413	CUAUUAGAAAAUUAAUUGCGAAAAAAAAAAAGGUUAGUCAUAUGUGCCUUUGCGGCGUCUUCCGCAGGAAGGACCCCUCUCUGCCAAUCACA<br>GAAGAGCUGGCUGUGCCUGUGCGGUACGCUAUCAGCAGCUGCGUCGGGUAAGGGACACGUCGUUAGUGACAAUCGUGGUUAGUUUUCGUAC<br>AGAAAGAUGACCUUAAUUGAUAAUUAAAGUAUAGAAAUGUCGUAUAGUAUAUUUUUCCCUUGCAUGGAAAAAACUGGGAUAAUGUGAUGA<br>UUCUCAUUAACAGAUUUACACUGGUAUUGAUACUCAUUUUGUUUCUGCUUCCUGUAGUACAUAAAAAGAUCUUGUAAAUAUUACGAUACU<br>CGUUAAAAUUUUCUUGAACGUAAUGUCGUACAAUUCUUGUUUGAGGGGUCG
GFP	339	AGGACGACGGCAACTACAAGACCCGCGCCGAGGTGAAGTTCGAGGGCGACACCCTGGTGAACCGCATCGAGCTGAAGGGCATCGACTTCAAGG<br>AGGACGGCAACATCCTGGGGCACAAGCTGGAGTACAACTACAACAGCCACAACGTCTATATCATGGCCGACAAGCAGAAGAACGGCATCAAGG<br>TGAACTTCAAGATCCGCCACAACATCGAGGACGGCAGCGTGCAGCTCGCCGACCACTACCAGCAGAACACCCCCATCGGCGACGGCCCCGTGCT<br>GCTGCCCGACAACCACTACCTGAGCACCCAGTCCGCCCTGAGCAAAGACCCCAACGAGA
